# Quantification of the Environmental Impact of Feeding Yeast Probiotic *Saccharomyces cerevisiae* Actisaf Sc 47 in Dairy Cow: A Life Cycle Assessment Approach

**DOI:** 10.3390/ani14152202

**Published:** 2024-07-29

**Authors:** Nizar Salah, Héloïse Legendre, Erika Paiva, Julie Duclos, Maxime Briche, Mariem Maaoui, Jasper Scholten, Céline Garat Boute

**Affiliations:** 1 Phileo by Lesaffre, 59520 Marquette-lez-Lille, France; h.legendre@phileo.lesaffre.com (H.L.); e.paiva@phileo.lesaffre.com (E.P.); j.duclos@phileo.lesaffre.com (J.D.); m.briche@phileo.lesaffre.com (M.B.); c.garatboute@phileo.lesaffre.com (C.G.B.); 2 Blonk Sustainability Tools, Groen van Prinsterersingel 45, 2805 TD Gouda, The Netherlands; mariem@blonksustainability.nl (M.M.); jasper@blonksustainability.nl (J.S.)

**Keywords:** yeast probiotic, life cycle assessment, dairy cow, yeast probiotic, Actisaf Sc 47

## Abstract

**Simple Summary:**

In this study, a life cycle assessment (LCA) was performed to quantify the environmental impacts of milk production in dairy cows with and without yeast probiotic supplementation. Faced with an ever-increasing population, livestock farming is in a paradoxical situation. Indeed, we went from a simple equation of how to increase animal performance to a more complex equation of how to increase performance and feed efficiency while reducing the environmental footprint as a pillar to guarantee more sustainable livestock systems for current and future generations, which is highly requested by all stakeholders in the chain including farmers, consumers, and society. Probiotics such as live yeast are commonly used to improve the performance and resilience of high-yielding dairy cows. In this study, we used a life cycle assessment (LCA) principle to quantify the environmental impact of a yeast probiotic, *Saccharomyces cerevisiae* (CNCM I-4407, 10^10^ CFU/g, Actisaf^®^ Sc 47), in dairy cows during three different trials. On average, during the period of supplementation, Actisaf Sc 47 reduced carbon footprint by 5%. Reported on a yearly scale including all lactation cycles, dry cows, and periods with and without supplementation, the use of Actisaf Sc 47 during the three trials reduced the carbon footprint, land use, water use, resource use, acidification, freshwater eutrophication, marine eutrophication, and terrestrial eutrophication by 2.9, 2.05, 2.47, 1.67, 2.28, 2.18, 2.14, and 2.28%, respectively. The supplementation of Actisaf Sc 47 showed very low contribution to the total carbon footprint, ranging from 0.005 to 0.016%.

**Abstract:**

Today, one of the major challenges of dairy farmers is to reduce their environmental footprint to establish more effective, efficient, and sustainable production systems. Feed additives such as yeast probiotics could potentially allow them to achieve these objectives through the improvement of milk production, feed efficiency, and ration valorization, hence mitigating the environmental impacts of milk production. In this study, the life cycle assessment (LCA) principle was performed to estimate the environmental impact of the production and supplementation of a commercial yeast probiotic (Actisaf Sc 47) in three trials performed in three different countries that are representative for around 50% of the milk production in Europe: France (French trial), United Kingdom (UK trial), and Germany (German trial). For each trial, two groups of animals were compared: control, without Actisaf Sc 47 supplementation, used as baseline; and experimental, with Actisaf Sc 47 supplementation at 5 or 10 g/cow/day. Different impact categories were analyzed for each group to calculate the impact of producing 1 kg of fat- and protein-corrected milk. An initial analysis was done only during the period of Actisaf Sc 47 supplementation and showed than the supplementation with Actisaf Sc 47 reduced, on average by 5%, the carbon footprint during the three trials. A second analysis was done via the extrapolation of all the data of each trial to an annual farm level, including the lactation period (305 days), dry period (60 days), and the period with and without Actisaf Sc 47 supplementation. Reported at a farm annual scale, the average reduction allowed by Actisaf Sc 47 supplementation was 2.9, 2.05, 2.47, 1.67, 2.28, 2.18, 2.14, and 2.28% of the carbon footprint, land use, water use, resource use, acidification, freshwater eutrophication, marine eutrophication, and terrestrial eutrophication, respectively. On average, the production of 1 kg of fat- and protein-corrected milk by using Actisaf Sc 47 was shown to improve environmental impacts compared to control. Regarding Actisaf Sc 47 production, the LCA showed that the production of 1 kg of Actisaf Sc 47 emitted 2.1 kg CO_2_ eq with a negligible contribution to total the carbon footprint of milk ranging from 0.005 to 0.016%. The use of Actisaf Sc 47 in dairy cows could then result in different positive outcomes: improving performance and efficiency while reducing the global carbon footprint.

## 1. Introduction

Climate change is widespread, rapid, and intensifying from year to year, and its consequences are increasingly visible and felt worldwide, including more and more heatwaves, floods, fires, and rising sea levels [1,2]. One of the main contributors to these changes is agriculture and, more particularly, livestock farming. Despite its contribution to the economy in different forms such as yielding milk, providing meat and by-products, and the creation of employment and wealth [3,4], the livestock sector contributes significantly to the most serious environmental problems through the use of land, water, and greenhouse gas emissions especially in ruminants via rumen fermentation [5]. Livestock emit various gases such as methane (CH_4_), nitrous oxide (N_2_O), and carbon dioxide (CO_2_) that impact the environment and contribute to global warming. Reported in terms of carbon footprint scales, these emissions accounted for 8.1 gigatons of CO_2_-eq per year from which cow’s milk production accounts for a large portion, contributing to 20 percent of the sector’s emissions [6,7,8]. Ruminants account for three-quarters of total CO_2_-equivalent (CO_2_-eq) emissions from the livestock sector. The global dairy sector alone emits 4% of the global anthropogenic GHG emissions. Hence, dairy farming needs to engage in environmental impact assessments [6]. To combat climate change and reduce emissions, concerted and collective action from all sector stakeholders is urgently required to ensure that existing and promising mitigation strategies are implemented. The need to reduce the sector’s emissions and its environmental footprint has indeed become ever more pressing in view of its continuing expansion to ensure food security while ensuring more efficient, resilient, and sustainable systems [7].

To deal with the situation, numerous efforts have been put in place, especially from a nutritional point of view. Thus, different feed additives have been developed to reduce methane indirectly by increasing feed efficiency and animal performance or directly by reducing methane production in the rumen. Among these additives, yeast probiotics are widely used in dairy cows, and Actisaf Sc 47 (*Saccharomyces cerevisiae* (CNCM I-4407, 10^10^ CFU/g, Actisaf^®^ Sc 47), produced and marketed by Phileo, is a very good example. Several studies have been conducted on this product under different conditions either through individual trials or through meta-analysis approaches and showed its positive effects on milk yield, milk quality, feed efficiency, feed valorization, digestibility, and rumen metabolism [9,10,11,12]. By increasing feed efficiency, the use of live yeast such as Actisaf Sc 47 could decrease the global environmental impact of milk production. However, to our knowledge, the combined effects of Actisaf Sc 47 on both milk performance and direct environmental parameters has not been previously studied. 

Our study was conducted to evaluate the global environmental footprint of a commercial yeast probiotic Actisaf Sc 47 (Phileo by Lesaffre, Marcq-en-Barœul, Lille, France) from its production process to farm performances using the life cycle assessment (LCA) approach according to ISO 14040 [13] and ISO 14044 standards [14].

## 2. Materials and Methods

**Figure 1:**
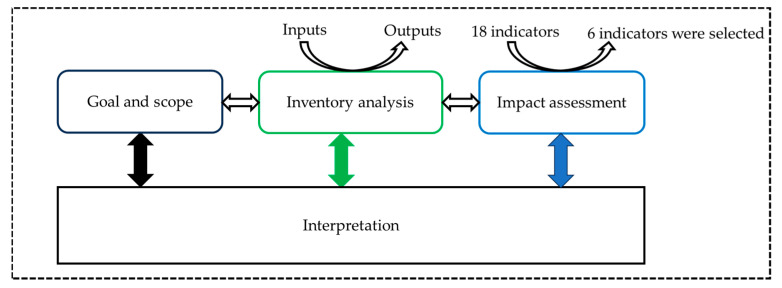
Flow diagram of the process of the LCA.

During our study, the ISO 14040 [13] and ISO 14044 [14] standards were used to implement a life cycle assessment (LCA) widely used as a reference methodology to evaluate the environmental performance of products, goods, and services. The Life Cycle Impact Assessment (LCIA) methodology refers to the recommendations and requirements given by the European Commission in the Product Environmental Footprint (PEF). In addition, the LCA used in this study followed Product Environmental Footprint Category Rules (PEFCR) for dairy products and animal feed [15,16]. Four steps were followed to establish the LCA: (1) definition of the goal and scope (including functional unit, and limits of the system); (2) the life cycle inventory (LCI) analysis (including the input and output data collection for all processes); (3) the life cycle impact assessment (LCIA); and (4) the life cycle interpretation (Figure 1). The LCA was submitted to an external critical review according to the standards ISO 14040 and ISO 14044. The critical review focused on the analysis of the goals and scope, analysis of the inventory, and the evaluation of the impacts and the interpretation. During the critical review, the consistency of the methods, the scientific and technical validity of the methods applied, the appropriateness of the data, the assessment of the interpretation, and the transparency and consistency of the study report were analyzed and commented on by three different specialists.

### 2.1. Goal and Scope 

The goal of this LCA study is to quantify the environmental impacts of Actisaf Sc 47 production at the Lesaffre factory and the environmental consequences of using it in dairy cows. Several impact categories were analyzed during our study, including carbon footprint, land use, water use, acidification, eutrophication, and fossil resource use. To achieve the objective, three different trials were used. During each trial, two groups of dairy cows were compared: a control group without any Actisaf Sc 47 supplementation and an experimental group with Actisaf Sc 47 supplementation at the level of 5 g or 10 g/cow/day (Table 1). Zootechnical performances obtained from each group such as intake, milk production, and feed efficiency were used to calculate the environmental impacts. The difference between the control and experimental groups was used to calculate the environmental impact of Actisaf Sc 47 for each trial separately to consider the location, the diet characteristics, and management system.

**Figure 2:**
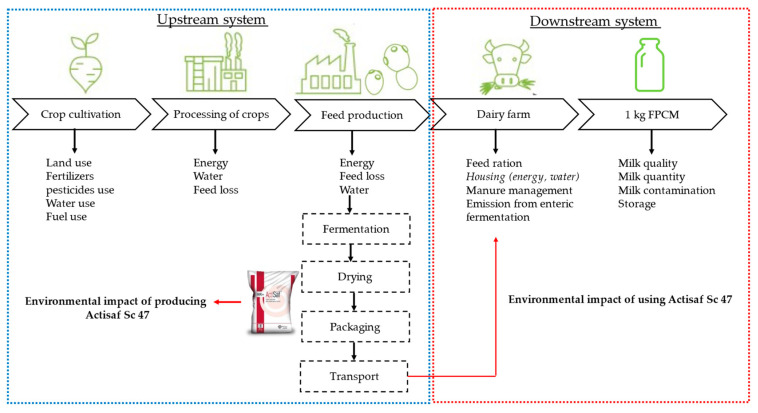
System boundary and functional units.

Two functional units were identified according to the system boundaries going from the cradle to the farm gate (Figure 2). The first unit corresponds to a production of 1 kg of Actisaf Sc 47 at the manufacturing level by the Lesaffre factory in France (Marcq en Baroeul), and the second unit corresponds to a production of 1 kg of fat- and protein-corrected milk (FPCM) at the farm level. The FPCM was calculated as follows, as defined by the European commission, 2018:

FPCM (kg) = milk yield (kg) × (0.1226 × Fat % + 0.0776 × protein % + 0.2534)

An uncertainty analysis required for comparative assertions was performed according to ISO 14044. The Monte Carlo method was performed to evaluate the uncertainty of measurements. SimaPro software 9.3 was used to run the Monte Carlo analysis. It takes a random value from the uncertainty distribution for each uncertain data input and calculates and stores the LCA results for this set of sampled values. The procedure was repeated an enormous number of times. Every time, SimaPro selects random values from the uncertainty distribution per data input, calculates the LCA results, and stores them. The stored LCA results for the defined number of iterations (generally 1000) form an uncertainty distribution for the final outcome. Given the small differences between the two systems (including and excluding Actisaf Sc 47) and the complex modeling, which does not clearly separate the paired uncertainties, it was not possible to use the Monte Carlo analysis results in this study. Therefore, the uncertainty analysis was performed by focusing on the key parameters that are relevant to the results. The focus was on general parameters, including enteric fermentation and climate change emissions and trial specific parameters including DMI, milk production, and methane emission. For general parameters, we used the uncertainty results available in the Intergovernmental Panel on Climate Change documentation (IPCC, 2006 [17] and IPCC, 2021 [18]. For trial-specific parameters, we used the results obtained during each trial. The two main parameters considered were the standard error (SE) to assess the representativeness of the samples and the *p*-value to evaluate the difference with *p* < 0.05 indicated as significant. 

### 2.2. System Boundary Definition

The system boundaries considered in the study comprised from cradle to Actisaf Sc 47 plant gate (upstream system) and from cradle to farm gate (downstream system) (Figure 2). The system boundaries defined what was included in the system and was therefore analyzed. At the same time, the system boundaries describe what was outside the system and therefore not included in this study. The upstream system includes crop cultivation, crop processing, feed production, indirect emissions linked to the use of fertilizers and pesticides, transport, fermentation, and drying to estimate the environmental impact of producing 1 kg of Actisaf Sc 47. The downstream system includes all the on-farm operations (e.g., animal feeding and care, milking procedures) and sources pertaining to forage production (e.g., arable land, agrochemicals, water). The transport of off-farm feeds, fossil fuels, and bedding materials and their emissions were also included in the assessment. Data related to feed cultivation and processing, transport, and non-agricultural feed ingredients were obtained from Agri-footprint 5.0 (Blonk consultant; 2019b [19] and Ecoinvent 3.6. The quantity of ingredients, quantity of energy, and the type of packaging used to produce Actisaf Sc 47 was provided by the company. Capital goods such as buildings, machines, and other basic infrastructure were excluded in line with the latest PEFCR guidelines. We chose these system boundaries to limit the complexity of our LCA. Furthermore, the different guidelines and PEFCRs are inconsistent on how they model the “after farm” impacts. The choice for these restricted boundaries implies that the “after farm” impact of some feed additives is not or incompletely modeled. 

### 2.3. Allocation Criterion

Allocation is used to distribute the overall environmental impacts of the different outputs. Even if milk is the major product of dairy activity, by-products such meat from calves and culled cows is an is an inevitable and valuable coproduct of dairy production. According to ISO 14044 [14], wherever possible, allocation should be avoided through subdividing a process into sub-processes or through system expansion. If this is not possible, allocation should be based on underlying physical relationships of the different products or functions, or alternatively, on other relationships, such as their economic value. In this study, economic allocation was applied at the cultivation stage and crop processing, in line with the PEFCR for feed or food producing animals [16]. The biophysical allocation criterion, which is extensively used in the dairy sector, was applied for two co-products as suggested by the dairy PEFCR [15]. The allocation criterion was calculated from the equation below:

$$AF=1-6.04\times \frac{Mmeat}{Mmilk}$$

where *AF* is the allocation factor of milk, *Mmeat* is the mass of the live weight of all animals sold, including bull, calves, and culled manure animals per year, and *Mmilk* is the mass of fat- and protein- corrected milk sold per year. The allocation of meat (*AM*) was then calculated from the equation below:

AM=1−AF

This procedure is based on the physical causality, accounting for the feed energy demand needed for producing milk and meat, respectively. A conservative approach was applied for the on-farm allocation. More environmental impacts were allocated to the system with Actisaf Sc 47 (more milk yield results in a higher allocation percentage).

### 2.4. Inventory Analysis and Input Data

Actisaf Sc 47 production was assessed from crop cultivation, raw material acquisition to the storage of the commercial probiotic at the plant. For the upstream system, specific data related to feed cultivation, processing and transport were obtained from Agri-footprint 5.0 [19] and Ecoinvent 3.6. All data linked to the different steps followed to produce Actisaf Sc 47 from the reception at the factory to the storage including energy consumption, water consumption, quantity of substrate, treatment, fermentation, drying, and packaging were provided by the company based on the LCA analysis for Actisaf production performed by EVEA in 2022.

For the downstream system, the effects of Actisaf Sc 47 on milk performances were obtained from three trials conducted in three different countries, each characterized by its own management system, rations, and animal potential. During each trial, two groups of lactating cows were compared: a control group without any Actisaf Sc 47 supplementation, and an experimental group with Actisaf Sc 47 supplementation at 5 to 10 g/d/cow (Table 1). During the French and UK trials, the supplementation started at calving and covered the peak of lactation; however, in the German trial, the supplementation started just after the peak of lactation. The difference between the two groups in each trial was used to quantify the environmental impact of Actisaf Sc 47. Other data related to zootechnical performances and feed characteristics, data linked to herd characteristics, energy input, water input, and bedding material were used as indicated in the dairy module in the Animal Production System footprint platform (APS-footprint from Blonk Sustainability tools [20]. These parameters were used to calculate the footprint per 1 kg of FPCM output from cows supplemented with Actisaf Sc 47 compared to un-supplemented cows. The footprint consisted of emissions from enteric fermentation and manure management such as methane and direct and indirect nitrous oxide (N_2_O) calculated with the Intergovernmental Panel on Climate Change (IPCC), and non-methane volatile compounds (NMVOC) and particulate matter (PM2.5 and PM10) calculated with air pollutant emission in the inventory guidebook from the European Environment Agency (EMEP/EEA). Emissions related to energy use and bedding material modeled with Ecoinvent energy and Agrifootprint were also used to calculate the final environmental footprint.

The experimental trials selected for the present study provided part of the data required for the LCA of dairy production. Zootechnical performances such as milk production, milk composition, dry matter intake, and feed efficiency obtained during each trial were provided to calculate the environmental impacts of animals in the control and experimental groups and quantify the potential effects of using Actisaf Sc 47 (Table 2). Non-available data such as energy consumption, manure management, and herd composition were provided from the dairy reference system in Blonk’s database. For each trial, a reference system was used that can be considered as an average farm corresponding to each country and embedded in the Agri footprint database V6. The duration of each trial does not cover the entire lactation, and zootechnical performances outside the period of the trials were not available. Milk production was therefore assumed to be equal to that of reference system used for each trial without any difference between the control and experimental groups. Dry matter intake outside the trial period was calculated according to the following equation:

$$FI\;outside\;trial\;period\;(kg)=\frac{FI\;during\;the\;trial\;\left(kg\right)\times MY\;outside\;trial\;period}{MY\;during\;trial\;period}$$

where *FI* = feed intake and *MY* = milk yield.

### 2.5. Life Cycle Impact Assessment

Data were analyzed using SimaPro 9.3 and the animal production system (APS) footprint tool [20]. A total of six indicators over the 18 as indicated by the European Commission were selected and calculated: carbon footprint, land use, water use, acidification, eutrophication, and fossil resource use.

## 3. Results

### 3.1. Environmental Characterization of Actisaf Sc 47 Production

The environmental impact categories of Actisaf Sc 47 production at the Lesaffre factory are presented in Table 3. In terms of climate change, LCA analysis showed that the production of 1 kg of Actisaf Sc 47 generated 2.10 kg CO_2_ eq. The land, water, and fossil fuel used to produce 1 kg of Actisaf Sc 47 was estimated at 54.78 Pt, 9.35 m^3^ deprivation eq and 52.21 MJ, respectively. Regarding eutrophication, the total potential contribution to freshwater eutrophication, marine eutrophication, and terrestrial eutrophication to produce 1 kg of Actisaf was 0.35 g P eq, 4.6 g N eq, and 0.06 mol N eq, respectively.

### 3.2. Environmental Characterization of Actisaf Sc 47 Application in Dairy Farms

**Figure 3:**
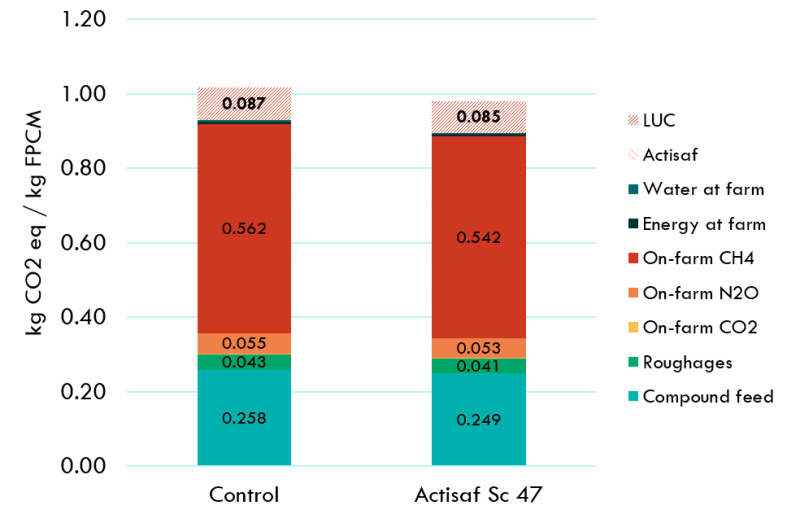
Contribution analysis to the carbon footprint of 1 kg FPCM produced with or without Actisaf Sc 47 during the French trial. LUC: land use change; FPCM: fat- and protein-corrected milk.

Due to the different conditions linked to the location, management system, and rations and animals used during each trial, the environmental impacts of the three farms were analyzed separately. For the French trial, the environment benefits that resulted from the supplementation of Actisaf Sc 47 in dairy cows compared to standard conditions without Actisaf Sc 47 supplementation are shown in Figure 3 and Table 4. Actisaf Sc 47 supplementation reduced the carbon footprint by 3.6% (1.016 vs. 0.98 kg CO_2_ eq/kg FPCM without and with Actisaf Sc supplementation, respectively) for a full farm including lactating cows, dry cows, and other animals and for a full year estimation including the period with and without Actisaf Sc 47 supplementation. Methane emissions from enteric fermentation and manure management, compound feed production, emissions from land use change (LUC), and N_2_O emissions from on-farm manure management were the categories that contributed most to the potential impacts, with contribution percentages of 55.33, 25.4, 8.61, and 5.42%, respectively. As indicated in Figure 3 and Table 4, the use of Actisaf decreased the impact of all the contribution categories. The environmental gain generated by Actisaf Sc 47 resulted mainly from better feed efficiency.

**Figure 4:**
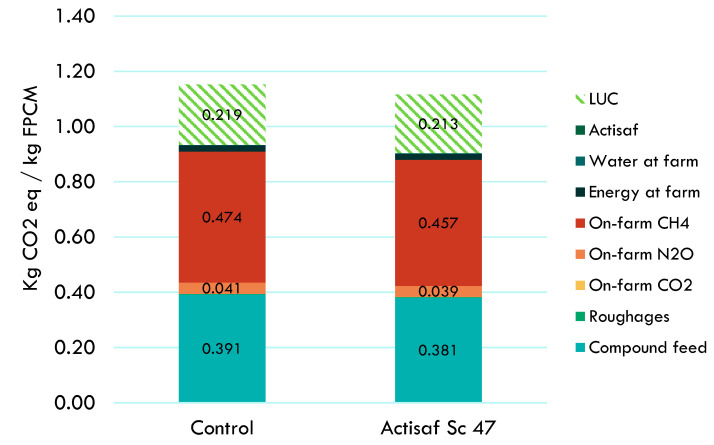
Contribution analysis to the carbon footprint of 1 kg FPCM produced with or without Actisaf Sc 47 during the UK trial. LUC: land use change; FPCM: fat- and protein-corrected milk.

For the UK trial, the environment benefits that resulted from the supplementation of Actisaf Sc 47 in dairy cows compared to standard conditions without Actisaf Sc 47 supplementation are shown in Figure 4 and Table 4. Actisaf Sc 47 supplementation reduced carbon footprint by 3.1% (1.152 vs. 1.116 kg CO_2_ eq/kg FPCM without and with Actisaf Sc supplementation, respectively) for a full farm including lactating cows, dry cows, and other animals and for a full year estimation including the period with and without Actisaf Sc 47 supplementation. Methane emissions from enteric fermentation and manure management, compound feed production, emissions from land use change, and N_2_O emissions from on-farm manure management were the categories that contributed most to the potential impacts, with contribution percentages of 41.17, 33.97, 18.97, and 3.52%, respectively. As indicated in Figure 4, the use of Actisaf decreased the impact of all the contribution categories. The environmental gain generated by Actisaf Sc 47 resulted mainly from better feed efficiency.

**Figure 5:**
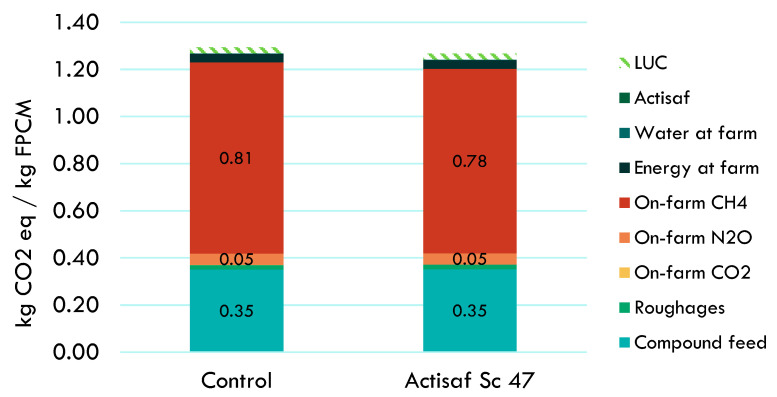
Contribution analysis to the carbon footprint of 1 kg FPCM produced with or without Actisaf Sc 47 during Germany trial. LUC: land use change; FPCM: fat- and protein-corrected milk.

For the German trial, the environment benefits that resulted from the supplementation of Actisaf Sc 47 in dairy cows compared to standard conditions without Actisaf Sc 47 supplementation are shown in Figure 5 and Table 4. Actisaf Sc 47 supplementation reduced the carbon footprint by 2.05% (1.29 vs. 1.27 kg CO_2_ eq/kg FPCM without and with Actisaf Sc supplementation, respectively) for a full farm including lactating cows, dry cows, and other animals and for a full year including the period with and without Actisaf Sc 47 supplementation. Methane emissions from enteric fermentation and manure management, compound feed production, emissions from land use change, and N_2_O emissions from on-farm manure management were the categories that contributed most to the potential impacts, with contribution percentages of 62.80, 27.10, 3.90, and 2.31%, respectively. As indicated in Figure 5, the use of Actisaf decreased the impact of all the contribution categories. The environmental gain generated by Actisaf Sc 47 resulted mainly from better feed efficiency.

Table 5 presents the mean environmental impacts of the control group without Actisaf Sc 47 supplementation and the experimental group with Actisaf Sc 47 supplementation and the potential benefits that resulted from using Actisaf in dairy cows. During the supplementation period, Actisaf Sc 47 reduced, on average, the carbon footprint by up to 5%; however, considering all lactation periods (305 days) and dry periods, the reduction was 2.9%. The extrapolation of all the data including lactation periods, dry periods, other non-milking animals, and periods with and without Actisaf Sc 47 to a yearly scale showed an average reduction of the carbon footprint by 2.9% (1.15 vs. 1.12 kg CO_2_ eq/kg FPCM without and with Actisaf Sc supplementation, respectively). The difference in carbon footprint reduction obtained during the period of Actisaf Sc 47 supplementation and extrapolated to a yearly scale can be explained by the fact that outside of the Actisaf supplementation period, all parameters such as intake, milk production, and feed efficiency were equal to the control. Compared to the control, supplementation with Actisaf Sc 47 reduced by 2.9, 2.05, 2.47, 1.67, 2.28, 2.18, 2.14, and 2.28% of the carbon footprint, land use, water use, resource use, acidification, freshwater eutrophication, marine eutrophication, and terrestrial eutrophication, respectively.

## 4. Discussion

Feeding the world’s population while minimizing the contribution of agriculture to climate change is one of the greatest challenges facing modern society. This challenge is particularly pronounced for dairy production where the carbon footprint of products and the mitigation costs are high relative to other food stuffs [21]. Calculating the environmental footprint is a fundamental way to measure, monitor, and improve the impact we have on the planet and to build more a sustainable production system with low emissions of greenhouse gases and fewer resources needed for any type of production. The life cycle assessment is a good tool used not only in agriculture but also across industries for quantifying the environmental impacts of a variety of specific products. As a key player in the market of probiotics and other yeast-based products and committed to supporting the sustainable transition of the agro-industry, Phileo by Lesaffre conducted a study to assess the environmental impact of Actisaf Sc 47, a yeast probiotic widely used in dairy cows. Indeed, three different dairy farms in Europe were used in our study to estimate the potential effects of producing 1 kg FPCM with and without using Actisaf Sc 47 on six different environmental categories: carbon footprint, land use, water use, resources use, acidification, and eutrophication.

The main results showed that the production of 1 kg of Actisaf Sc 47 emitted 2.10 kg of CO_2_ eq in which 1.26 kg of CO_2_ eq was emitted during the fermentation process. The high contribution of fermentation processes may be explained by the high substrate demand of this step. The production of 1 kg of Actisaf Sc 47 required 54.75 Pt of land and 9.35 m^3^ of water. This is mainly due to the use of land to cultivate sugar cane and sugar beet and water during both fermentation process and irrigation to produce molasses as the main substrate from sugar cane and sugar beet. Packaging was the subsystem that contributes especially to land use, and this may be due do the pallet. The reuse of pallets could be a solution to reduce the impact of packaging in land use and the global carbon footprint. Regarding drying, natural gas was the main contributor for climate change, resource use, and acidification, while potato starch was the main contributor for marine eutrophication and water use. Regarding transportation, our study revealed that the environmental impacts generated by truck transport was very negligible due to the short distances, even if transport by truck seems to have a higher environmental impact than other transportation solutions [22].

Our results showed that the main factors responsible for environmental impacts in the three farms either for the control or the Actisaf Sc 47 group are methane emissions from enteric fermentation and manure management, compound feed production, emissions from land use change, and N_2_O emissions from on-farm manure management. By using an LCA approach, [23] identified methane from enteric fermentation, nitrous oxide from fertilizers used for on-farm and off-farm feed production, and methane plus nitrous oxide from manure management as main contributors to the global carbon footprint.

To the best of our knowledge, there are no previous published studies evaluating the global environmental impact of a yeast probiotic in dairy cows. Our results showed that using 5 g to 10 g of Actisaf Sc 47 in dairy cows reduced the carbon footprint CO_2_ eq during the three trials. A more beneficial effect was observed during the supplementation period during which the carbon footprint was reduced by 5% compared to 2.9% when extrapolated to a yearly scale. This difference can be attributed to the fact that outside the periods of Actisaf Sc 47 supplementation, all animals are considered equal in terms of performance and feed efficiency. In dairy systems, this impact category is mainly affected by the methane emissions from enteric fermentation, feed production, and N_2_O emission [24]. We observed that the use of Actisaf Sc 47 decreased the impact of the contribution of these three categories during the different trials. Since methane emissions and feed, including roughages and compound feeds, represented a big part of the total carbon footprint explained by kg CO_2_ eq per 1 kg FPCM produced, it seems clear that the improvement of performance and feed efficiency is a key to reducing the environmental impact of dairy cows during the three trials. The positive effect of Actisaf Sc 47 supplementation on feed efficiency explains its beneficial effect on climate change. The cows that received Actisaf Sc 47 ingested less feed to produce the same amount of milk, which explains their better efficiency. A favorable correlation was observed between CH_4_ emission per kg of milk produced and feed efficiency, whereby more efficient cows may be emitting less CH_4_ per kg milk, which would explain the reduced environmental impacts permitted by Actisaf Sc 47 [25]. The use of Actisaf Sc 47 in dairy cows is well documented either in vitro or in vivo by using simple individual trials or meta-analysis approaches. By its effect on the optimization of the rumen environment and metabolism, Actisaf Sc 47 increased milk quantity and quality, feed efficiency, digestibility, and feed valorization [9,10,11,12,13,14,15,16,17,18,19,20,21,22,23,24,25,26,27,28]. A recent meta-analysis showed that supplementation with 5 g of Actisaf Sc 47 increased MY and energy-corrected milk (ECM) by 1.75 and 2.45 kg, respectively [10]. The analysis of data collected from 27 different trials showed that the supplementation of dairy cows with 5 g of Actisaf Sc 47 increased MY, ECM, and FCM by 1.32, 1.6, and 1.53 kg, respectively [9]. Previous studies have used LCA to assess the environmental impact of feed additives on ruminants, swine, broiler chickens, and turbot and attributed their beneficial effect on the carbon footprint to their positive effects on feed efficiency [29,30].

For terrestrial and freshwater acidification, our results showed that the use of Actisaf Sc 47 reduced acidification for the three trials. In dairy cattle, the main contributors to terrestrial acidification are emissions of ammonia and nitrous oxide, which partly originate from animal excreta and manure management and application [31,32]. These two parameters are linked to the rumen environment and nitrogen use efficiency. Studies indicate that nitrogen use efficiency in dairy cows is low and may range from less than 20% to a theoretical maximum of approximately 45%. The remaining nitrogen is excreted in urine and feces, which contribute to acidification, with ammonia (NH_3_) and nitrous oxide (N_2_O) as main contributors [31,32,33]. Improving nitrogen (N) efficiency is a way to reduce ammonia and nitrous oxide and consequently acidification [34]. For this purpose, high condensed tannins foods could be administered, which increases the rumen by-pass protein [35]. Indeed, according to [36], high rumen degradable protein can determine asynchrony between amino acids and energy availability, causing higher NH_3_ production in the rumen and consequently in animal excretions. The mechanism by which Actisaf Sc 47 could reduce acidification may be attributed to its positive effect on nitrogen use efficiency. Studies have shown that the use of Actisaf Sc 47 stimulates the growth and activity of fibrolytic bacteria [26,27,28,29,30,31,32,33,34,35,36,37]. Rumen fibrolytic bacteria have a strong preference for ammonia [38] as a nitrogen source, which increases ammonia utilization for microbial protein synthesis and overall nitrogen utilization and consequently reduction in urinary nitrogen losses, as well as a reduction in ammonia emissions [39]. Ref. [40] observed a positive correlation between ammonia concentration in the rumen and ammonia concentration in manure. A positive correlation between live yeast supplementation and rumen microbial protein synthesis has been demonstrated by [41], who indicated that the addition of *saccharomyces cerevisiae* increased the use of ammonia in the rumen and a reduction of its level in manure and protein waste.

Water and terrestrial eutrophication have become a worldwide environmental problem in recent years. Livestock manure is largely composed of organic compounds including nitrogen (N) and phosphorus (P), which results in water and soil eutrophication [42]. The biological mechanism by which Actisaf Sc 47 could reduce eutrophication is unknown and has not been described previously. We hypothesize that Actisaf Sc 47 improves nitrogen efficiency and stimulates the growth of phytase-producing bacteria, such as *Selenomonas ruminantium*, *Megasphaera elsdenii,* and *Prevotella* sp., which increases phosphorus use efficiency and consequently reduces phosphorus excretion in manure [43,44]. However, it would be interesting to investigate the influence of feeding Actisaf Sc 47 to dairy cows on nitrogen and phosphorus balance and the characteristics of farm manure to assess if these aspects could also further benefit its overall environment effects. To the best of our knowledge, no specific data analyzing the environmental footprint of a yeast probiotic distributed to dairy cows were found, but studies have compared different production management systems: conventional, organic, mountain, and plain [25,26,27,28,29,30,31,32,33,34,35,36,37,38,39,40,41,42,43,44,45,46]. These authors demonstrated a negative correlation between feed efficiency, carbon footprint, land and water use, acidification, and eutrophication. During the three trials analyzed in our study, Actisaf Sc 47 led to an increase in feed efficiency, which explains its global beneficial effects.

As shown in Figure 2, Figure 3 and Figure 4, the contribution of using Actisaf Sc 47 on the total carbon footprint is low and was minimal compared to the environmental impact of the three production systems. This may be explained by the low inclusion rate in feed (5–10 g/d), the positive effects of Actisaf Sc 47 on performance, feed efficiency, low impact of the fermentation-based process to produce Actisaf Sc 47, and most environmental impact categories. In dairy cows, different feed additives have been evaluated for their environmental impact by using the LCA approach [47]. According to [47], the general use of additives can lead to up to 10% improvement in the environmental footprint due primarily to their positive effect on animal performances, feed efficiency, and greenhouse gas emissions. The use of Actisaf Sc 47 allowed us to achieve a reduction ranging from 2.05 to 3.6 by analyzing the entire lactation cycle and up to 5% reduction by just analyzing the supplementation period. The difference from the value of 10% mentioned by [47] can be explained by the proper mode of action of each additive, the dose, the direct effect on environmental parameters such as methane and ammonia, or indirect effects such as improving animal health, fertility, and efficiency. As mentioned previously, no published LCA was carried out to evaluate the environmental impact of yeast probiotics in dairy cows. The use of Actisaf Sc 47 as a fermentation-based solution might be of high interest due to the lower environmental impact. The main limitations of this study were firstly the assumptions that were made for the missing data points based on reference dairy systems representing the country’s average milk production. Secondly, the extrapolation of trial data to annual farm-level data means that the effect of Actisaf was only included during the trial period. The days of lactation outside the trial scope were assumed equal to the control for all zootechnical parameters including DMI, MY, FE, milk quality, and emissions. This diluted the reduction from approximately 5% to approximately 3%.

## 5. Conclusions

The positive effects of Actisaf Sc 47 in rumen metabolism, milk performance, feed efficiency, and economic gain are well known and studied compared to their effect on the environment. An LCA study on Actisaf Sc 47 production and its use in dairy cows was herein performed to assess its environmental impact by analyzing different categories. The LCA was submitted for an external critical review according to ISO 14040 and ISO 14044 standards. Based on an LCA performed by EVEA in 2022, the production of 1 kg of Actisaf Sc 47 emitted 2.1 kg CO_2_ eq while using 54.78 Pt of land and 9.35 m^3^ deprivation eq of water. In dairy cow production, the supplementation of Actisaf Sc 47 to dairy cows added 0.005 to 0.016% to the total carbon footprint, which is very negligible compared to other categories. On the other hand, the use of Actisaf Sc 47 reduced the carbon footprint by 5% during the period of supplementation, which covers both peak and mid-lactation. Reported on the annual farm level, including the lactation period, dry period, and the period with and without Actisaf supplementation, the use of Actisaf Sc 47 in three different dairy farms reduced the carbon footprint by 2.9%. On average, the use of Actisaf Sc 47 during the three trials reduced the carbon footprint, land use, water use, resource use, acidification, freshwater eutrophication, marine eutrophication, and terrestrial eutrophication by 2.9, 2.05, 2.47, 1.67, 2.28, 2.2, 2.14, and 2.28%, respectively. These beneficial effects may be attributed to the positive effect of Actisaf Sc 47 on performance and feed efficiency. Other benefits, such as the improvement of reproduction performances, health, and manure quality, were not included in this study, suggesting a higher impact reduction. Other trials with supplementation during the entire lactation period are suggested and could confirm a positive effect over a shorter period.

## Figures and Tables

**Table 1:** Overview of selected trials used to calculate the environmental footprint of Actisaf.

	French Trial	UK Trial	German Trial
Title	Effect of Actisaf supplementation on performances, ruminal fermentation characteristics and health parameters of peripartum dairy cows	Effects of Actisaf live yeast on performance of high yielding dairy cow	Impact of probiotic live yeast Actisaf Sc 47 on digestive, metabolic and performance parameters in high yielding dairy cows during summertime
Organization	INRAe Toulouse, France	University of Nottingham, UK	Training and research center, Futterkamp, Germany
Duration of supplementation	108 days	120 days	92 days
Stage of lactation	Day 1 to day 108	Day 1 to day 120	Day 109 to day 197
Year	2018	2019	2021
Number of cows	10	50	72
Dose of Actisaf Sc 47	5 g/cow/d	10 g/cow/day	5 g/cow/day

**Table 2:** Zootechnical performances during all trials and extrapolation to annual values.

	French Trial		UK Trial		German Trial	
	Control	Actisaf Sc 47	Control	Actisaf Sc 47	Control	Actisaf Sc 47
Duration of the trial	108	108	120	120	92	92
MY (kg/d)	37.9	42.9	47.5	50.1	34.1	34
MY during the trial (kg)	4093.2	4633.2	5700	6012	3137.2	3128
MY of reference system (kg/year)	7373	7373	12,500	12,500	7748	7748
MY 109–305 d of lactation	4206	4206	(-)	(-)		
MY 121–305 d of lactation	(-)	(-)	6263	6236		
MY day 1–108 and 202–305	(-)	(-)	(-)	(-)	5502	5502
Total annual milk (kg)	8802	8836	11,963	12,275	8639.2	8630
Fat %	4.29	4.33	3.9	3.97	3.84	3.81
Protein %	3	3.06	3.25	3.23	3.35	3.35
Total annual FPCM (kg/cow)	8403	9028	11,768	12,161	8502	8462
FI (kg DM/cow/d)	21.9	23.4	23.9	24	21.9	20.9
FI during the trial (kg DM/cow)	2365.2	2527.2	2868	2880	2015	1923
FI out of the trial period (kg DM/cow)	2427	2427	3151	3151	3533	3533
Estimated FI during the dry period (kg/cow)	600	600	600	600	600	600
Annual FI (kg DM/cow) ^1^	5392.2	5554.2	6619	6631	6148	6056
GAEI (MJ/cow)	99,464	102,453	122,122	122,341	113,430	111,733
FE (kg FPCM/kg DMI)	1.75	1.83	1.94	2.02	1.53	1.6
CH_4_ (kg/d)			0.467	0.462		
CH_4_ (kg/kg ECM)			0.01	0.0095		
CH_4_ (kg/kg DMI)			0.0197	0.0194		

MY: milk yield; FI: feed intake; FPCM: fat- and protein- corrected milk; FE: feed efficiency; GAEI: gross annual energy intake; ^1^ calculated through cross multiplication with milk yield; ECM: energy-corrected milk.

**Table 3:** Environmental characterization of 1 kg Actisaf Sc 47 product.

Environmental Impact Category	Unit	Actisaf Sc 47
Climate change	Kg CO_2_ eq	2.10
Land use	Pt	54.78
Water use (deprivation potential)	m^3^ depriv eq	9.35
Resource use (fossil)	MJ	52.21
Freshwater eutrophication	g P eq	0.35
Marine eutrophication	g N eq	4.60
Terrestrial eutrophication	mol N eq	0.06

**Table 4:** Contribution analysis results for carbon footprint of three trials with and without Actisaf Sc 47 supplementation. results are expressed in kg co2 equivalents per kg fat and protein corrected milk.

	French Trial		UK Trial		German Trial	
	Control	Actisaf Sc 47	Control	Actisaf Sc 47	Control	Actisaf Sc 47
Total	1.016	0.980	1.152	1.116	1.29	1.27
Feed compounds	0.258	0.249	0.391	0.381	0.35	0.35
Roughages	0.043	0.041	0.002	0.002	0.02	0.02
On farm CO_2_	0.001	0.001	0.000	0.000	0.00	0.00
On-farm N_2_O	0.055	0.053	0.041	0.039	0.05	0.05
On farm CH_4_	0.562	0.542	0.474	0.457	0.81	0.78
Energy at Farm	0.007	0.006	0.023	0.022	0.04	0.04
Water at Farm	0.003	0.003	0.002	0.002	0.002	0.002
Actisaf Sc 47	0.0000	0.0001	0.0000	0.0002	0.0000	0.0001
Land use change	0.087	0.085	0.219	0.213	0.03	0.03

**Table 5:** Contribution of using Actisaf Sc 47 in dairy cows to the environmental footprint reported on a yearly scale.

	Environmental Impact Category	Control	Actisaf	Diff
Average of the three trials	CC (kg CO_2_ eq)	1.15 × 10^0^	1.12 × 10^0^	−2.9%
	LU (Pt)	4.96 × 10^1^	4.86 × 10^1^	−2.05%
	WU (m^3^ depriv)	2.45 × 10^−1^	2.39 × 10^−1^	−2.47%
	RU fossil (MJ)	2.75 × 10^0^	2.70 × 10^0^	−1.67%
	TFWAC (mol H+ eq)	1.94 × 10^−2^	1.90 × 10^−2^	−2.28%
	FE (g P eq)	1.05 × 10^−4^	1.03 × 10^−4^	−2.18%
	ME (g N eq)	8.81 × 10^−3^	8.62 × 10^−3^	−2.14%
	TE (mol N eq)	8.66 × 10^−2^	8.46 × 10^−2^	−2.28%

CC: climate change; LU: land use; WU: water use; RU: resource use; TFWAC: terrestrial and freshwater acidification; FE: freshwater eutrophication; ME: marine eutrophication; TE: terrestrial eutrophication.

## Data Availability

Data contained within the article.
